# 

**Published:** 1846-12

**Authors:** 


### WESTERN JOURNAL

OF

### MEDICINE AND SURGERY.

### EDITORS.

---

DANIEL DRAKE, M.D.,

PROFESSOR OF PATHOLOGY AND THE PRACTICE OF MEDICINE IN THE MEDICAL INSTITUTE OF LOUISVILLE:

LUNSFORD P. YANDELL, M.D.,

PROFESSOR OF CHEMISTRY AND PHARMACY IN THE SAME:

THOMAS W. COLESCOTT, M.D.

### INDEX TO VOL. VI.

[NEW SERIES.]

---

A

Abscess of lungs opening externally, 452.
Acid, prussic, new test for, 156.
Aconitum napellus in neuralgia, 353
Address to the Medical Profession, 530.
Ague, Graves on relapse periods of 78.
Alabama, med. topography of, 460.
Alkalies in cutaneous diseases, 257.
Amputation, statistics of, 1.
Analysis of blood, 211, 267.
Anatomy, pathological, 519.
Anecdote, medical, 544.
Aneurism, false, 122, 218.
Aneurism treated by compression, 153.
Aneurism spontaneously cured, 533.
Animal chemistry, 235.
Authorship of Vestiges of Creation, 92.
Army, British. flogging in, 346.
Arsenic, poisoning with, 350, 538.
Asphyxia from drowning cured, 163
Atlas of anatomy, 434.
Atrophy of a muscle, 459.

B

Balsam of Peru in diabetes, 351.
Barytes in scrofula, 521.
Berkley's case of double consciousness, 204.
Biliary calculus, 82.
Bilious fever, 93, 107.
Blood in asthma, fever, and epilepsy, 211.
Blood in bodies killed by strangling, 527.
Blood, analysis of, 267.
Boling on amputation in private practice, 1.
Boling on fever, 255.
Bowels, electricity in obstructions of. 81.
Brain, tubercles of, 476.
Braithwaite's Retrospect, 437.
Brocchieri water, 534.
Bromine and its preparations, 523.
Broussais, sketch of, 341.
Buck on the blood in disease, 211.
Buffalo medical journal, 89.
Buffalo medical school, 457.
Burn, deformity from, relieved, 147.

C

Cæsarian operation, 456.
Calculus, biliary, 82.
Calculus, urinary, from a female, 152.
Caloric, Metcalfe on, 19.
Cataract, treatment of. 538.
Castor oil, how to give it, 78.
Chemistry of the four seasons, 566.
Chemistry animal, 236.
Chemistry, by Fownes, 313.
Chlorosis in adults, 259.
Cholera, Asiatic, 83, 540, 542, 543, 544.
Circular, Dr. Harris's, 364.
Circular, National Institute, 366.
Clymer on fevers, 135.
Cod-liver oil in strumous affections, 522.
Colchicum in neuralgia, 445.

Cold water treatment, death from, 351.
Coley on diseases of children, 430.
Composition of air, &c., 520.
Compound fracutre of cranium, 535
Congenital dropsy, 458.
Consumption, statistics of, 438.
Convention, medical, of Ohio, 146.
Consciousness, double, 204.
Constipation, nervous, treatment of, 538.
Copland's dictionary prac. med., 152
Correspondence, foreign, 85, 127, 217, 453, 479.
Cranium, fracture of, 535.
Creosote in diarrhœa, 448.
Cutaneous diseases, alkalis in, 257.

D

Dawson on the tongue, 277.
Deafness, 164.
Delirium tremens, 542.
Diabetes mellitus, balsam Peru in, 351.
Diarrhœa, creosote in, 448.
Diarrhœa of infants, 450.
Dickson on quinine, 185.
Digestion, 541.
Dissector, by Horner, 437.
Draper's chemistry, 431.
Dropsy, congenital, 458.
Drug-shop of Galen, 266.
Dunglison's med. dictionary, 182.
Dunglison's physiology, 433.
Dupuytren, sketch of, 269.

E

Ellis's medical formulary, 434.
Electro-magnetism in obstructed bowels, 81.
Electro-magnetism in intermittent fever, 367.
Electro-magnetism in poisoning by opium. 446.
England, medical examinations in, 82.
Epilepsy successfully treated, 16.
Ergotine, 534.
Erysipelas, Velpeau on, 454.
Examinations, medical, in England, 82.

F

Faraday's lecture, 229.
Femoral hernia, case of, 132.
Fee's account of a shower of maggots, 183.
Fevers, 93, 107, 135, 255, 299.
Flogging in the British army, 346.
Forbes on homœopathy, 181, 260.
Foree's report of a case of aneurism, 122.
Foreign correspondence, 85. 127, 217, 453, 479.
Fracture of the cranium, 535.
Fry on epidemic bilious fever, 93.

G

Galen's drug-shop, 266.
Galen's writings, 439.
Gangrene, spontaneous, dry, 148.
Garden of plants, 480.
Gold in dyspepsia, 484.
Gordon on congestive fever, 299.
Gordon's private medical teaching, 89.
Graves on relapses in agues, 78.
Grant, Prof., as a lecturer, 130.
Griffith's chemistry, 516.

H

Harper on bilious fever, 107.
Harris's med. topography, 460.
Hasse's pathological anatomy, 510.
Hemorrhage, opium in, 525.
Hemostatic virtues of Brocchieri water and ergotine, 534.
Hernia, femoral, 132.
Heurnius's institutes, 305.
Hippocrates's writings, 436, 485.
Hooping cough, 352.
Hot-water in asphyxia, 163.
Homœopathy, Forbes on, 181, 26[illegible].
Honey-bee in strangury, 268.
Hospitals in London, 222.
Hunterian museum, 224.
Hydropathy, death from, 351.
Hydrophobia, remedy for, 484.

I

Infants diseased from milk, 157.
Inflammations, articular, 543.
Insanity, when most common, 176.
Institution, Royal, 229.
Intermittent fever, 367, 441.
Indigestion, gold in, 484.
Iodide of iron, process for, 457.

Iodine in oil, 482.
Iron, iodide of, process for, 457.
Iron, sulphate of, in excessive perspiration, 528.

J

Johnson's natural philosophy, 89.
Journal of medicine, Southern. 92.

L

Larkins on mania a potu, 474.
Larrey, sketch of, 344.
Lead, poisoning by, 540.
Liebig, a billet-doux to, 356.
Liquids, spherical state of, 233.
Leucorrhœa, 367.
Liston's personal appearance, 128.
London hospitals, 222.
Louisville, health of, 179, 274.
Louisville law school, 274.
Louisville summer med. school, 375
Louisville University, 179, 546.
Lungs, abscess of, opening externally, 452.

M

Mammary glands, premature development of, 458.
Mania, puerperal and a potu, 474.
Magneto-electricity in paralysis, 442
Magneto-electricity in poisoning by opium, 446.
Magendie's introductory lecture, 73.
Maggots, a shower of, 183.
Metcalfe on caloric, 19.
Medical miscellany, 92.
Medical societies, 177, 180, 181.
Medical schools, statistics of, 529.
Medical profession, address to, 530.
Membrana tympani, perforation of 164.
Milk as a cause of disease in infants, 157.
Mosul, heat of, 438.
Muriate of barytes in scrofulous affections, 521.
Murphy, Prof., on females, 219.

N

Neuralgia, aconitum in, 353.
Neuralgia, colchicum in, 445.
Nervous constipation, treatment of, 538.

O

Obstruction of bowels, electricity in, 81.
Oil as a solvent for iodine, 483.
Ointment for ophthalmia, 483.
Ophthalmic ointment, 483.
Opium poisoning and electricity, 447
Opium in hemorrhage, 525.
Organic matters, how preserved, 349
Osborne's case of epilepsy, 16.

P

Paralysis, magneto-electricity in, 442.
Paraphymosis, 79.
Placenta prævia, 258.
Poisoning by arsenic, 538.
Poisoning by lead-shot, 540.
Potash, nitrate of, 541.
Powers, 'the grinder,' 218.
Prize essay, Louisiana, 164.

Q

Quackery, 347.
Quackery in Cincinnati, 359.
Quain on strictures of urethra, 221.
Quinine, 185, 460.
Quinine applied endermically, 172.

R

Royal institution, 229.

S

Scarlatina in Tennessee, 276.
Scrofula, employment a cause, 265.
Scrofula, Sutton's essay on, 269.
Scrofula, Phillips on, 436.
Scrofulous affections, muriate of barytes in, 521.
Simon's animal chemistry, 236.
Sims on trismus nascentium, 91.
Sneed's case of tubercles, 476.
Society, medical, in Tennessee, 177
Strangury, new remedy for, 268.
Statistics of medical schools, 529.
Stramonium cigars, 525.
Strumous affections, cod-liver oil in, 522.
Suit for malpractice, 122.
Sulphate iron for excessive perspiration, 528.
Sulphate of quinine, 185.
Surgery, dental, 547.

Sulphate of quinine endermically, 172.
Sulphate of quinine, œdema after use, 460.
Surgery, Velpeau's. 50.

T

Tennessee medical society, 177, 180.
Tight lacing, effects of, 166.
Tongue in disease, 277.
Trismus nascentium, 91, 173.
Trismus simulating hydrophobia, 149.
Treatment of warts, 525.
Treatment of cataract, 538.
Treatment of nervous constipation, 538.
Tubercles of the brain, 476.
Typhoid fever, 358.

U

University of London, course of study in, 87.
University of Louisville, 179, 546.
Upas tree, 528.

V

Velpeau's surgery, 50.
Velpeau on erysipelas, 454.
Vision, acute, a case of, 456.

W

Warts, treatment of, 525.
Westbrook on scarlatina, 276.
Wistar's anatomy, 432.
White swelling, treatment of, 440.

Y

Yandell, D. W.'s letters, 127, 217, 453, 479.

